# Redox biology response in germinating *Phaseolus vulgaris* seeds exposed to copper: Evidence for differential redox buffering in seedlings and cotyledon

**DOI:** 10.1371/journal.pone.0184396

**Published:** 2017-10-05

**Authors:** Inès Karmous, Rafael Trevisan, Ezzeddine El Ferjani, Abdelilah Chaoui, David Sheehan

**Affiliations:** 1 Plant Toxicology and Molecular Biology of Microorganisms, Faculty of Sciences of Bizerta, Zarzouna, Tunisia; 2 Nicholas School of the Environment, Duke University, Durham, North Carolina, United States of America; 3 College of Science, Khalifa University of Science and Technology, Abu Dhabi, United Arab Emirates; 4 School of Biochemistry and Cell Biology, University College Cork, Cork, Ireland; University of Nebraska-Lincoln, UNITED STATES

## Abstract

In agriculture, heavy metal contamination of soil interferes with processes associated with plant growth, development and productivity. Here, we describe oxidative and redox changes, and deleterious injury within cotyledons and seedlings caused by exposure of germinating (*Phaseolus vulgaris* L. var. soisson nain hâtif) seeds to copper (Cu). Cu induced a marked delay in seedling growth, and was associated with biochemical disturbances in terms of intracellular oxidative status, redox regulation and energy metabolism. In response to these alterations, modulation of activities of antioxidant proteins (thioredoxin and glutathione reductase, peroxiredoxin) occurred, thus preventing oxidative damage. In addition, oxidative modification of proteins was detected in both cotyledons and seedlings by one- and two-dimensional electrophoresis. These modified proteins may play roles in redox buffering. The changes in activities of redox proteins underline their fundamental roles in controlling redox homeostasis. However, observed differential redox responses in cotyledon and seedling tissues showed a major capacity of the seedlings’ redox systems to protect the reduced status of protein thiols, thus suggesting quantitatively greater antioxidant protection of proteins in seedlings compared to cotyledon. To our knowledge, this is the first comprehensive redox biology investigation of the effect of Cu on seed germination.

## Introduction

Heavy metal contamination of agricultural soils can interfere with plant physiological processes associated with normal growth and development [1], and may induce alterations within plant cells [2, 3]. One of the underlying causes of tissue injury following exposure of plants to heavy metals is overproduction of reactive oxygen species (ROS) [3–5], which can react directly with biomolecules (lipids, DNA and proteins) leading to biochemical alterations [6].

At the cellular level, plants have evolved both enzymatic and non-enzymatic defense mechanisms against deleterious effects of heavy-metal-induced oxidative stress [7]. Non-enzymatic antioxidant molecules include ascorbate (AsA), reduced glutathione (GSH), carotenoids, alkaloids, tocopherols, proline, and phenolic compounds (flavonoids, tannins, and lignin) which act as low M_r_ free radical scavengers [8, 9]. Enzymatic antioxidant defenses include superoxide dismutase (SOD; EC 1.15.1.1) [10], glutathione peroxidases (GPX; EC 1.11.1.9), peroxidase (POX; EC 1.11.1.7) and catalase (CAT; EC 1.11.1.6), as well as the enzymes of the ascorbate-glutathione cycle (Asc-GSH) [11], ascorbate peroxidase (APX; EC 1.11.1.11), glutathione reductase (GR; EC 1.6.4.2), monodehydroascorbate reductase (MDHAR) and dehydroascorbate reductase (DHAR). Additionally, enzymatic functioning of the Asc-GSH, involving successive oxidation and re-reduction of Asc, GSH and NAD(P)H [12], depends on reducing power [13, 14] supplied directly at the expense of photosynthesis or via secondary NAD(P)H recycling dehydrogenase activities; glucose-6-phosphate dehydrogenase (G6PDH; EC 1.1.1.4), 6-phosphogluconate dehydrogenase (6PGDH; EC 1.1.1.44), malate dehydrogenase (MDH; EC 1.1.1.37) and isocitrate dehydrogenase (threo-D-isocitrate: NADP^+^ oxidoreductase, EC 1.1.1.42) [15].

In plants, modulation of cellular redox homeostasis involves three principal systems: glutaredoxin (Grx)/GSH/GR, thioredoxin/NADPH/NADPH-dependent thioredoxin reductase (EC 1.6.4.5) (Trx/NADPH/ NADPH-dependent thioredoxin reductase (NTR); EC 1.8.1.9) [16–18], and ferredoxin-NADP oxido-reductase (FNR, EC 1.18.1.2). Functioning of these redox system components is based on their redox activity [19, 20]. In germinating seeds, their roles have previously been investigated [21, 22]. Indeed, Trx has been shown to be involved in extensive changes in the redox state of major storage proteins, the thiols of which are converted from the oxidized (–S–S–) form to reduced (–SH) forms [21]. They also play a role in activation of proteases and inactivation of protease inhibitors [20–22]. Under normal conditions, the intracellular redox state is predominantly reducing, but processes such as oxidative stress, notably under abiotic- and biotic-induced stress, can shift the redox balance toward an oxidizing state [23–25]. Regulation of the redox status of proteins, including the sulfhydryl groups of cysteine, can act as a "switch" for the activity of proteins involved in specific signaling events and in cell cycle control [26].

Some of the recognized effects of Cu toxicity on bean seeds are: (1) alteration of germinative metabolism [27, 28]; and (2) disruption of the capacity of the ubiquitin-proteasome pathway to eliminate oxidatively-damaged proteins [29]. Besides, in cells, Cu exists in either a reduced (Cu^+^) or an oxidized (Cu^2+^) state, which makes Cu a redox-active metal by the induction of electron-transfer reactions. This redox-activity can also promote the generation of reactive oxygen radicals and affect every category of macromolecule. On these grounds, the present work aimed to shed more light on the mechanism of Cu-induced toxicity and on the cell defense response in bean cotyledons and seedlings. In particular, we are interested in elucidating changes in antioxidative enzymes (SOD, CAT, APX, POX and GPX) and enzymes of NAD(P)H-recycling dehydrogenases (G6PDH, 6PGDH and MDH) under Cu-induced stress. In addition, effects of Cu on the coenzyme pattern, NAD(P)H oxidase (EC 1.6.99.6; EC 1.6.99.3) activity and redox components (Grx, GR, Trx, NTR, Fd, FNR and peroxiredoxin (Prx; EC 1.11.1.15) were investigated.

## Materials and methods

### Germination and copper treatment conditions

Seeds of the bean (*Phaseolus vulgaris* L. var. soisson nain hâtif) were germinated at 25 ± 1.5°C in the darkness in the presence of H_2_O or 200 μM CuCl_2_, according to Karmous et al. [27]. Whole seedlings and cotyledons were collected, respectively, at days 3 and 9.

### Measurement of oxidative indicators

H_2_O_2_ levels were measured according to Sergiev et al. [30]. Carbonyl and thiol groups were determined according to the methods of Reznick and Packer [31] and Ellman [32], respectively. Measurements were performed using 0.5 mL of cotyledon or seedling extract in a total reaction volume of 2 mL.

### Enzyme assays

Cotyledons (6) or seedlings (12) were homogenized at 4°C with 25 mM potassium phosphate buffer (pH 7.0), (1:5, w/v), containing 5 mM sodium ascorbate, followed by centrifugation for 30 min at 20,000 × g. The resulting supernatant was considered as soluble enzymatic fraction. Protein concentrations in the cotyledon or seedling extract were evaluated by the method of Bradford [33], using bovine serum albumin as standard protein. SOD activity was measured according to Mishra and Fridovich [34]. The enzyme assay mixture (2 mL) contained 1.88 U mL^-1^ catalase, 62.5 mM sodium carbonate/bicarbonate (pH 10.4), 125 μM EDTA and 20 μL of the cotyledon or seedling extract. SOD activity was estimated at 490 nm, using epinephrine as standard. CAT activity was measured according to Aebi [35]. The enzyme assay mixture (2 mL) contained 10 mM H_2_O_2_ in 25 mM phosphate buffer (pH 7.0) and 100 μL of the cotyledon or seedling extract (diluted 10-fold). CAT activity was estimated by monitoring the decrease in absorbance of H_2_O_2_ reduction at 240 nm (ε = 36 × 10^−6^ M^-1^.cm^-1^). APX (EC 1.11.1.11) activity was measured according to Nakano and Asada [36]. The reaction mixture (2 mL) contained 0.5 mM ascorbate, 5 mM H_2_O_2_, 0.1 mM EDTA and 100 μL of the cotyledon or seedling extract. APX activity was determined by following the decrease in the absorbance of ascorbate at 290 nm (ε = 2.8 × 10^−3^ M^-1^.cm^-1^). POX activity was measured according to Fielding and Hall [37]. The reaction mixture (510 μL) contained 10 mM H_2_O_2_ in 25 mM potassium phosphate buffer (pH 7.0), 9 mM guaiacol and 10 μL of the cotyledon or seedling extract (diluted 5-fold). POX activity was estimated by the increase in absorbance of oxiguaiacol at 470 nm (ε = 26.6 M^-1^.cm^-1^). GPX activity was measured according to Nagalakshmi and Prasad [38]. The reaction mixture (520 μL) contained 50 mM phosphate buffer (pH 8.0), 100 mM NaCl, 1 mM GSH, 2.5 mM H_2_O_2_, 0.5 mM NADPH, 1 U GR and 20 μL of the cotyledon or seedling extract. Oxidation of NADPH was followed by measuring the decrease in absorbance at 340 nm (ε = 6.22 × 10^3^ M^-1^.cm^-1^, [17]). G6PDH and 6PGDH activities were measured in the medium (500 μL): 50 mM Tris-HCl buffer (pH 7.9), 5 mM MgCl_2_ and 0.2 mM NADP^+^, containing, respectively, 5 mM glucose-6-phosphate and 0.5 mM 6-phosphogluconate and 20 μL of the cotyledon or seedling extract. Production of NADPH was determined by measuring the increase in the absorbance at 340 nm (ε = 6.22 × 10^3^ M^-1^.cm^-1^, [20]). MDH activity was measured in the medium (500 μL): 400 mM hydrazine sulfate (pH 9.0), 500 mM glycine, 2.5 mM NAD^+^, 50 mM malate sodium, 10 mM MgCl_2_ and 10 μL of the cotyledon or seedling extract. The production of NADH was evaluated by measuring the increase in the absorbance at 340 nm (ε = 6.22 × 10^3^ M^-1^. cm^-1^, Bergmeyer et al. [39]). Activities of Trx and NTR were measured in the reaction mixture (500 μL): 50 mM Tris-HCl, pH 8.1, 100 μM DTNB and 20 μL of the cotyledon or seedling extract, and containing 0.2 mM NADPH and 30 μg mL^-1^ reduced Trx (NTR assay) or 15 mg mL^-1^ NADPH and 0.1 μM NTR (Trx assay). The reduction of dithio-bisnitrobenzoate (DTNB) was determined by measuring the increase in the absorbance at 412 nm (ε = 13.6 × 10^3^ M^-1^.cm^-1^,[40]). Grx activity was performed by HED reduction [41] in the medium (500 μL): 50 mM Tris-HCl (pH 8.0), containing 0.2 mM NADPH, 0.5 mM GSH, 0.5 U mL^-1^ GR (Sigma), 0.5 mM hydroxyethyl disulfide HED and 20 μL of the cotyledon or seedling extract. The oxidation of NADPH was measured at 340 nm (ε = 6.22 × 10^3^ M^-1^. cm^-1^). GR activity was determined according to Foyer and Halliwell [12], by following the rate of NADPH oxidation as a decrease in absorbance at 340 nm. The assay mixture (500 μL) contained 0.2 mM NADPH, 0.5 mM oxidized glutathione (GSSG) in 50 mM phosphate buffer (pH 7.0) and 20 μL of the cotyledon or seedling extract. Fd and FNR activities were assayed according to Green et al. [42]. The reaction mixture (500 μL) contained 20 μL of the cotyledon or seedling extract, 50 mM Tris-HCl (pH 7.8), 40 μM cytochrome C, 250 μM NADPH, 2 μM spinach leaf ferredoxin (FNR assay) or 0.1 μM FNR (Fd assay). The reduction of cytochrome C was monitored by the increase in absorbance at 550 nm (ε = 19.1 × 10^−3^ M^-1^.cm^-1^). Prx activity was measured in the medium (500 μL): 50 mM potassium phosphate (pH 7.0), containing 150 μM NADPH, 1 mM GSH, 0.5 U GR, 50 μM Grx, 1 mM H_2_O_2_ and 20 μL of the cotyledon or seedling extract. Oxidation of NADPH was monitored as a decrease in absorbance at 340 nm (ε = 6.22 × 10^3^ M^-1^.cm^-1^) [41]. Activities of NADPH oxidase and NADH oxidase were measured using 20 μL of the cotyledon or seedling extract in a total reaction mixture of 500 μL, containing 100 mM sodium acetate (pH 6.5), 1 mM MnCl_2_, and 0.5 mM *p*-coumaric acid, containing 0.2 mM NADPH (NADPH oxidase assay) or 0.2 mM NADH (NADH oxidase assay). Oxidation of NAD(P)H was measured as a decrease in absorbance at 340 nm (ε = 6.22 M^-1^.cm^-1^[43]).

### Coenzyme extraction and measurement

Reduced (NADPH and NADH) and oxidized (NADP^+^ and NAD^+^) forms of coenzyme were extracted according to the method of Zhao et al.[44], respectively, in 0.1 M NaOH and 0.1 M HCl, followed by centrifugation at 20,000 × g at 10 min at 4°C. Twenty μL of cotyledon or seedling extract in 500 μL total reaction volume were used for quantification according to the procedures described by Matsumura and Miyachi [45].

### Protein carbonyls and thiols

Proteins were extracted by homogenization (1:5, w/v) of cotyledons or seedlings in 10 mM Tris-HCl, pH 7.2, 500 mM saccharose, 1 mM EDTA, 150 mM KCl and 1 mM PMSF. After centrifugation at 20,000 ×g for 1 h at 4°C, supernatant was collected.

Protein carbonyls (CO) and thiols (SH) were labelled, respectively, at a final concentration of l mM with fluorescein-5-thiosemicarbazide (FTSC) and 0.2 mM 5’-iodoacetamide fluorescein (IAF). After 2 h incubation at 37°C for 150 min in the dark, proteins were precipitated with an equal volume of 20% TCA and centrifuged at 20,000 ×g for 3 min at 4°C. The pellets were then resuspended and washed three times with 100% ethanol/ethyl acetate (1:1) and 96% acetone, respectively, for CO and SH groups. Pellets obtained were resuspended in Tris-HCl 0.5 M pH 6.8, glycerol 10%, SDS 0.5% and bromophenol blue, then applied to 1D SDS-PAGE gels (12%, 120 V) [46] (Mini-PROTEAN system, Bio-Rad). Gels were scanned in a Typhoon Trio Scanner 9400 (Control v5.0 + variable Mode Imager-RA 501: PRT<I/06/004, GE Healthcare, UK; excitation, 490–495 nm; emission, 515–520 nm). Protein-associated fluorescence intensity (arbitrary units, AU) was analyzed using Quantity One image analysis software (BioRad, Hercules, CA, USA). Gels were then stained with Colloidal Coomassie Brilliant Blue G250 [47], scanned with a calibrated densitometer GS-800 (BioRad, Hercules, CA, USA) and the total OD was measured and normalized by the previously-used software.

For 2D gels, proteins were separated according to their pI (first dimension: isoelectric focusing IEF), then according to their molecular weight (second dimension: sodium dodecyl sulphate-polyacrylamide gel electrophoresis; SDS-PAGE). Proteins were first rehydrated in 5 M urea, 2 M thiourea, 2% CHAPS, 4% ampholyte (Pharmalyte 3–10, Amersham-Pharmacia Biotech, Little Chalfont, Bucks, UK), 1% Destreak reagent (Amersham-Pharmacia Biotech), and trace amounts of bromophenol blue, and then immobilized in 7 cm IPG strips pH 3–10 of dimension 70×3×0.5 mm and linear gradient (NL) (GE Healthcare Immobiline^™^ Dry Strip IPG, Bio-Sciences AB, Bio-Rad, Hercules, CA, USA), for the separation of a final volume of 125 μl. Proteins were focused on a Protean IEF Cell (Bio-Rad) for at least 15 h at room temperature, according to the following steps: (1) a linear voltage increase until 250 V for 15 min, (2) 10,000 V for 2 h (50 μA/ strip), (3) focusing at 20,000 V, and (4) hold at 500 V. Following IEF, strips were equilibrated for 20 min in equilibration buffer; 6 M urea, 0.375 M Tris, pH 8.8, 2% SDS, 20% glycerol, containing 2% DTT and then for 20 min in equilibration buffer containing 2.5% iodoacetamide. IPG strips were then loaded onto 12% SDS-PAGE gels (PROTEAN Plus Dodeca Cell Bio-Rad). After protein separation, gels were scanned for fluorescence as described above and then stained with Colloidal Coomassie Brilliant Blue R-250 followed by densitometry scanning. Normalization of FTSC- and IAF-labeled protein spots and Coomassie-staining intensity was performed using Progenesis SameSpots Software (Ref: S/No.62605/3787; Nonlinear USA Inc/2530 Meridian Parkway/ 3rd Floor Durham/ NC 27713/ USA) as per the manufacturer’s instructions. Fluorescence spots were normalized to protein intensity for the same gel revealing increased fluorescence.

### Statistical analysis

All experiments were performed at least in triplicate. Values are means ± standard error SE, of three technical and five biological replicates. These were compared for significance of differences at p < 0.05 using the ANOVA test followed by Student’s t test analysis. Images of 2D gels were subjected to landmarking alignment so that corresponding spots were matched with each other. This models protein spots mathematically as a 3D Gaussian distribution and determines maximum absorption after raw image correction and background subtraction. Spot intensities were normalized to make the total density in each gel image equal, and quantitative and qualitative analyses were performed. The protein spots were detected automatically and then edited manually to remove streaks, speckles, and artifacts. Two D gels were replicated at least three times and the results reported as means ± SD. Analyses of variance (one-way ANOVA) followed by Tukey’s *post hoc* multiple comparison tests were performed using the software package Statistica 8.0 to compare Cu-treated tissues with controls. Statistically significant differences between all spots in 2D gel image were established at p<0.05 and assessed using Student’s t test.

## Results

### Effects of copper on seed germination

**Fig 1:**
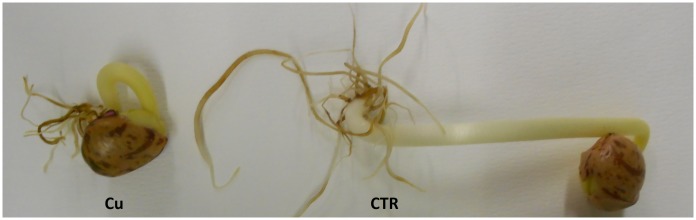
Growth of bean (*Phaseolus vulgaris* L. var. soisson nain hâtif) seedlings germinated in the presence of distilled water or 200 μM CuCl_2_.

**Fig 2:**
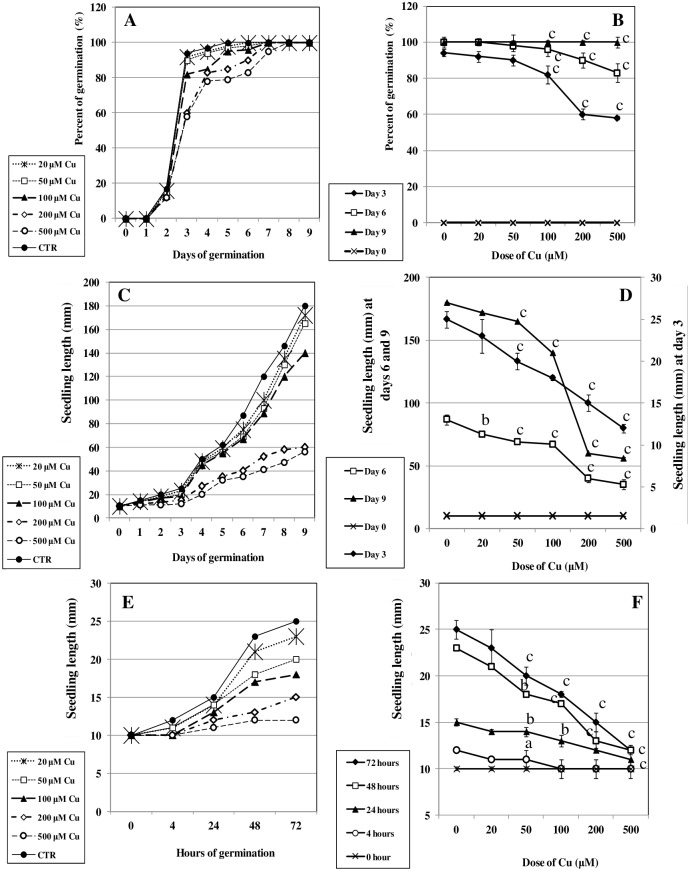
Time courses of (A, B) germination rate and (C, D, E, F) seedling length in bean seeds in the absence (CTR) and in the presence of CuCl_2_ (concentrations 20, 50, 100, 200, 500 μM). Letters indicate significant differences compared with the respective control sample (a: p < 0.05, b: p < 0.01 and c: p < 0.001).

Cu strongly inhibited germination of bean seeds, as evidenced by decreased growth of the Cu-treated seedlings over 9 days (Fig 1). A two-day delay in germination was evident in Cu-treated seeds (Fig 2A and 2B). However, the seedling length showed drastic decrease with increasing Cu concentration (Fig 2C–2F). Nevertheless, for the present study, we chose 200 μM Cu as our working concentration and days 3 and 9 for, respectively, seedlings and cotyledons.

### Response of antioxidant enzyme systems to copper-induced stress

**Fig 3:**
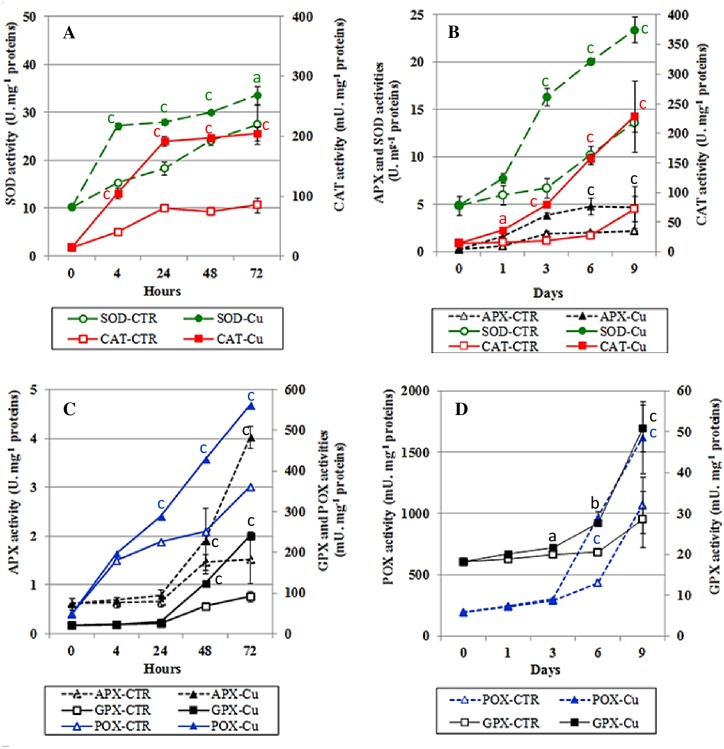
Enzymatic activities of SOD, CAT and peroxidases (APX, GPX and POX) in (A, C) seedlings and (B, D) cotyledons of bean seeds during germination in the presence of distilled water (CTR) or 200 μM Cu. Values are means ± SE (n = 5). Letters indicate significant differences compared with the respective control sample (a: p < 0.05, b: p < 0.01 and c: p < 0.001).

A significant increase of H_2_O_2_ content was observed in Cu-treated cotyledon extracts although no significant increase was evident in seedling extracts after 3 days’ treatment (Table 1). In S1 Appendix, we also recorded an increase in MDA levels in both tissues after exposure to Cu. Hence, we were interested to ascertain the mechanisms by which bean seeds respond to Cu-induced stress. Indeed, marked enhancement of the antioxidant enzymatic activities; SOD, CAT and peroxidases (APX, GPX and POX) in seedlings (Table 1) and cotyledons (Table 1) were evident after Cu treatment. This increase was significant for all antioxidant enzymes (except SOD and APX in cotyledons), as compared to controls. In addition, time courses of enzyme activities suggested that, in seedlings, SOD and CAT activities increased after only 4 hours of germination while POX, APX and GPX increased after 24 hours (Fig 3). In cotyledons, SOD, CAT and APX activities increased from the first day of germination, with more significant activation at days 3, 6 and 9 (Fig 3). However, GPX and POX showed increased activities after day 3.

**Table 1:** Level of H_2_O_2_ and activities of antioxidant enzymes in the seedlings after 3 days (a) and cotyledons after 9 days (b) of germinated bean seeds in the presence of H_2_O (CTR) or 200 μM Cu.

Parameters	Unit	CTR	Cu
H_2_O_2_	(a) μmol. g^-1^ fresh weight	0.90±0.13	1.27±0.02
	(b) μmol. g^-1^ fresh weight	1.53±0.24	3.10±0.32 *
SOD	(a) U. mg^-1^ proteins	27.47±4.15	33.56±1.93 *
	(b) U. mg^-1^ proteins	13.64±2.58	23.42±1.34 *
CAT	(a) mU. mg^-1^ proteins	85.32±12.26	204.36±11.08 ***
	(b) U. mg^-1^ proteins	72.83±21.02	229.20±83.49 *
APX	(a) U. mg^-1^ proteins	1.52±0.48	4.02±0.23 ***
	(b) U. mg^-1^ proteins	2.23 ± 0.05	4.68 ± 2.20
POX	(a) mU. mg^-1^ proteins	360.69±51.04	561.65±27.58 ***
	(b) mU. mg^-1^ proteins	1069.68 ± 227.44	1622.57± 295.70 *
GPX	(a) mU. mg^-1^ proteins	91.35±25.76	240.18±31.33 ***
	(b) mU. mg^-1^ proteins	28.66 ± 6.93	50.96 ± 5.64 **
G6PDH	(a) mU. mg^-1^ proteins	28.46±5.29	104.22±27.99 **
	(b) mU. mg^-1^ proteins	6.71 ± 2.54	13.63 ± 1.18 **
6PGDH	(a) mU. mg^-1^ proteins	137.35±25.87	289.67±39.81 ***
	(b) mU. mg^-1^ proteins	33.53 ± 7.48	67.84 ± 5.67 ***
MDH	(a) mU. mg^-1^ proteins	138.48±38.13	364.29**±**42.81 ***
	(b) U. mg^-1^ proteins	1.05 ± 0.02	1.97 ± 0.01

Values are means ± SE (n = 5). Asterisks indicate significant differences compared with the respective control sample

* p < 0.05,

** p < 0.01

*** p < 0.001

Upon Cu treatment, activities of NAD(P)H-independent dehydrogenases, notably, G6PDH, 6PGDH and MDH were significantly enhanced in both cotyledons and seedlings (Table 1). These biochemical observations led us to examine changes in protein redox status in response to Cu exposure, as well as possible relationships between protein thiol management and thiol-dependent enzymatic redox systems.

### Redox changes under copper-induced stress

Levels of both CO and -SH groups were higher in Cu-treated seedlings whilst, in cotyledons, an increase in CO level versus a net decline in level of protein -SH was observed (Table 2). This suggested that protein thiol status was affected by oxidation due to Cu in both organs. In addition, when compared to respective controls, cotyledons of Cu-treated seeds showed a significant decrease in Trx activity, but no significant variation in Grx activity and a marked increase in GR and NTR activities (Table 3). However, in seedlings, a significant increase in the activities of NTR and Trx was evident with no significant increase in GR and Grx activities in the presence of Cu (Table 3). These data suggest that, as a consequence of Cu-induced stress, there is a more protective effect of Grx/GR and Trx/NTR systems on protein thiols in seedlings than in cotyledons. Curiously, the Fd/FNR system was highly stimulated in seedlings whilst it was inhibited in cotyledons (Table 3). Prx activity also increased in both seedlings and cotyledons, as compared with controls, which may implicate this enzyme in Cu defense.

**Table 2:** Levels of protein carbonyl and thiol groups in the seedlings (3 days-old) and the cotyledons (9 days-old) of germinated bean seeds in the presence of H_2_O (CTR) or 200 μM Cu.

Parameters	Tissues	Unit	CTR	Cu
-CO	Seedlings	μmol mg^-1^ proteins	7.37±0.15	9.24±0.09 ***
	Cotyledons	nmol mg^-1^ proteins	7.96±1.89	15.12±0.30 **
-SH	Seedlings	nmol mg^-1^ proteins	90.90±18.40	292.83±19.98 ***
	Cotyledons	nmol mg^-1^ proteins	65.11±4.52	35.74±3.14 *

Values are means ± SE (n = 4). Asterisks indicate significant differences compared with the respective control sample

* p < 0.05,

** p < 0.01

*** p < 0.001

**Table 3:** Activities of redox enzymes in the seedlings (3 days-old) and the cotyledons (9 days-old) of germinated bean seeds in the presence of H_2_O (CTR) or 200 μM Cu.

Parameters	Tissues	CTR	Cu
NTR	Seedlings	0.68±0.23	1.36±0.29 *
	Cotyledons	2.07±0.89	4.00±0.56 *
Trx	Seedlings	2.10±0.37	6.51±1.79**
	Cotyledons	1.56±0.29	1.33±0.31
GR	Seedlings	12.09±2.28	18.40±1.26**
	Cotyledons	14.89±2.26	15.30±6.07
Grx	Seedlings	18.34±3.62	26.02±6.1*
	Cotyledons	5.10±1.56	4.81±2.01
FNR	Seedlings	16.34±3.20	42.28±7.78 ***
	Cotyledons	9.37±3.62	4.12±1.26 *
Fd	Seedlings	25.91±8.09	50.53±5.37 **
	Cotyledons	5.55±1.94	2.36±2.78
Prx	Seedlings	67.18±19.2	282.02±41.72 ***
	Cotyledons	79.57±4.38	100.00±12.56 *

Values are means ± SE (n = 5). NTR activity was expressed in U/mg proteins in cotyledons and mU/mg proteins inseedlings. Activities of Trx, GR, Grx, FNR, Fd and Prx were expressed in mU/mg proteins in both cotyledons and seedlings. Asterisks indicate significant differences compared with the respective control sample

* p < 0.05,

** p < 0.01

*** p < 0.001

To assess potential contributions of coenzyme forms in response to Cu-induced stress, possible changes in total quantities of nicotinamide coenzymes (oxidized form + reduced form) were examined. The enzymatic activities responsible for oxidation of the reduced forms of coenzyme were also measured. A net increase in total coenzyme levels was found in both cotyledons and seedlings (Table 4). The redox ratio of coenzymes (NADP^+^ /NADPH and NAD^+^ /NADH), as well as NADPH oxidase activity increased significantly in seedlings, whilst only the NAD^+^ /NADH ratio and NADH oxidase activity increased significantly in cotyledons. These findings suggested elevated levels of oxidized coenzyme forms (NADP^+^ and NAD^+^) in response to Cu treatment, as compared with controls (Table 4).

**Table 4:** Activities of NAD(P)H oxidases and redox ratios of coenzymes in the seedlings (3 days-old) and the cotyledons (9 days-old) of germinated bean seeds in the presence of H_2_O (CTR) or 200 μM Cu.

Parameters	Tissues	CTR	Cu
Total coenzymes	Seedlings	91.62±1.38	107.14±1.43 ***
	Cotyledons	1870.61±22.27	2953.85±36.85 **
NADP^+^ /NADPH	Seedlings	0.68±0.04	2.02±0.19 ***
	Cotyledons	1.57±0.24	1.91±0.35
NAD^+^ /NADH	Seedlings	6.04±0.64	15.20±0.10 ***
	Cotyledons	4.54±0.60	6.35±0.75 ***
NADPH oxidase	Seedlings	694.57±51.74	841.67±31.45 *
	Cotyledons	368.89±100.95	429.59±43.85
NADH oxidase	Seedlings	397.52±127.33	540.23±128.32
	Cotyledons	384.14±103.19	579.81±72.24 *

Values are means ± SE (n = 5). Total coenzymes (oxidized and reduced forms) were expressed in nmol/g fresh weight, and activities of NAD(P)H oxidases were expressed in mU/mg proteins. Asterisks indicate significant differences compared with the respective control sample

* p < 0.05,

** p < 0.01

*** p < 0.001

### Changes in protein thiol and carbonyl groups

**Fig 4:**
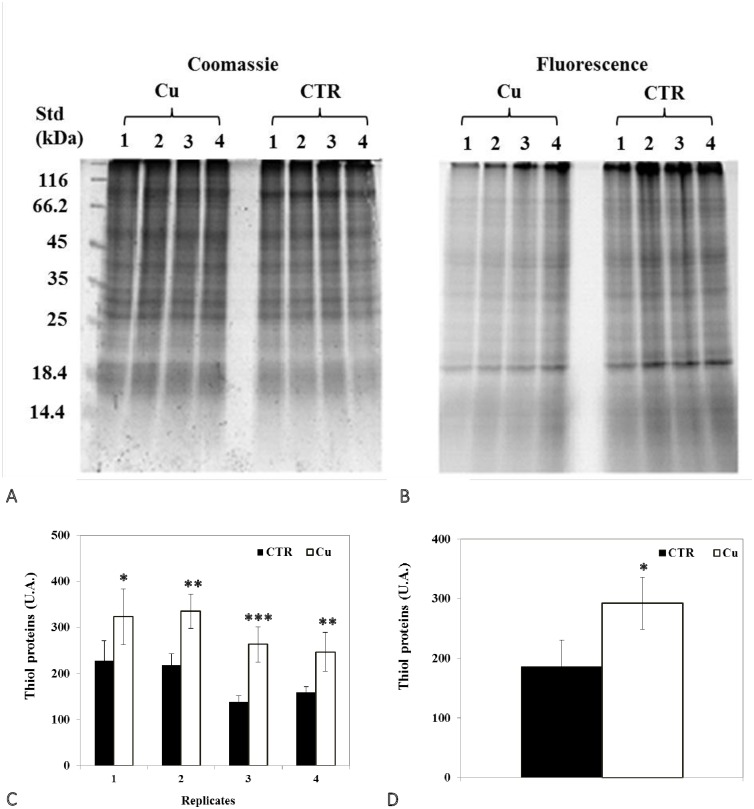
Representative images of 1DE gels of proteins (100 μg) in seedlings of bean seeds germinated for 3 days. (A) In the presence of distilled water (CTR) or (B) 200 μM Cu. Gels were stained with Coomassie G-250 (scanned with GS-800 calibrated densitometer) and with IAF labeling (scanned with Typhoon 9400 scanner). Total optical densities for each lane obtained from IAF staining were normalized with those from Coomassie G-250 staining of the same gel. (C, D) Levels of proteins containing thiol groups. Values shown are (C) means of 4 biological replicates (±SD) numbered from 1 to 4, and (D) means of 4 technical replicates (±SD). Each measurement was performed in an extract obtained from several seedlings. Analyses were performed using ANOVA, student’s T test; *p<0.05, **p<0.01, ***p<0.001.

**Fig 5:**
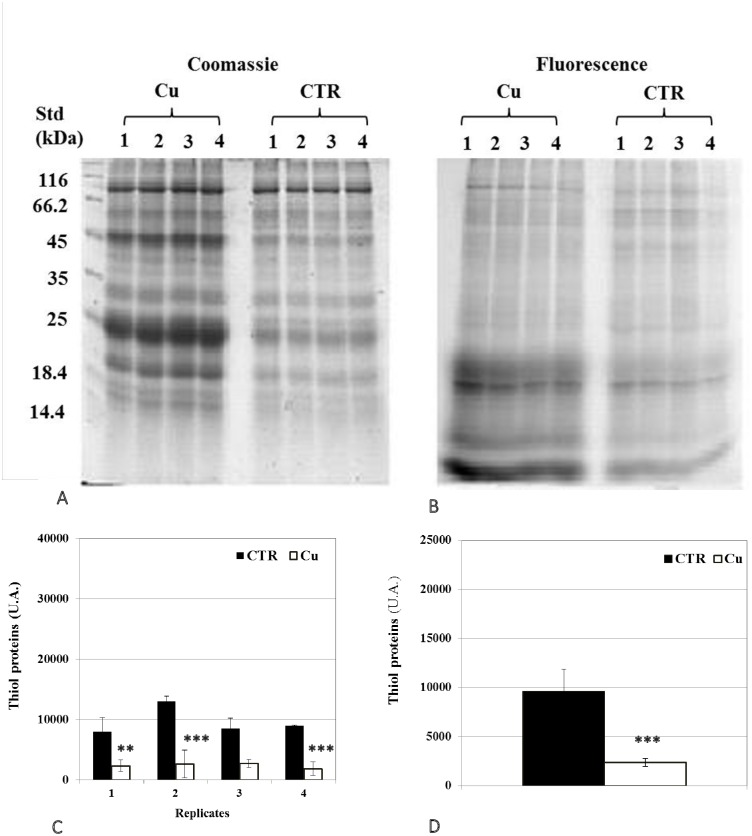
Representative images of 1DE gels of proteins (150 μg) in cotyledons of bean seeds germinated for 9 days in the presence of (A) distilled water (CTR) or (B) 200 μM Cu. Gels were stained with Coomassie G-250 (scanned with GS-800 calibrated densitometer) and with IAF labeling (scanned with Typhoon 9400 scanner). Total optical densities for each lane obtained from IAF staining were normalized with those from Coomassie G-250 staining of the same gel. (C, D) Levels of proteins containing thiol groups. Values shown are (C) means of 4 biological replicates (±SD) numbered from 1 to 4, and (D) means of 4 technical replicates (±SD). Each measurement was performed in an extract obtained from several cotyledons. Analyses were performed using ANOVA, student’s T test; **p<0.01, ***p<0.001.

**Fig 6:**
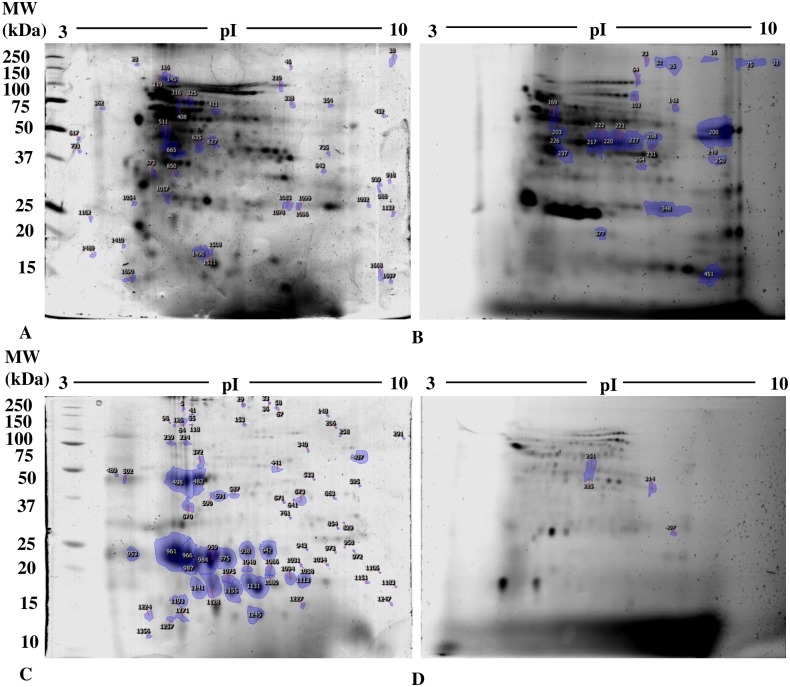
Profiles of the expression of proteins containing thiol groups in (A, B) seedlings and (C, D) cotyledons of bean seeds germinated for 9 days in the presence of distilled water (CTR) or 200 μM Cu. Proteins (800 μg) were labeled with IAF and separated by 2-D SDS-PAGE. Figures show spots of interest in representative gels from (A, C) colloidal Coomassie Brilliant G-250 staining (scanned with GS-800 calibrated densitometer) and (B, D) IAF labeling (scanned with Typhoon 9400 scanner; 800 PMT). Numbers correspond to spots of p<0.05 and Fold induction >1.5 (spots identified).

**Fig 7:**
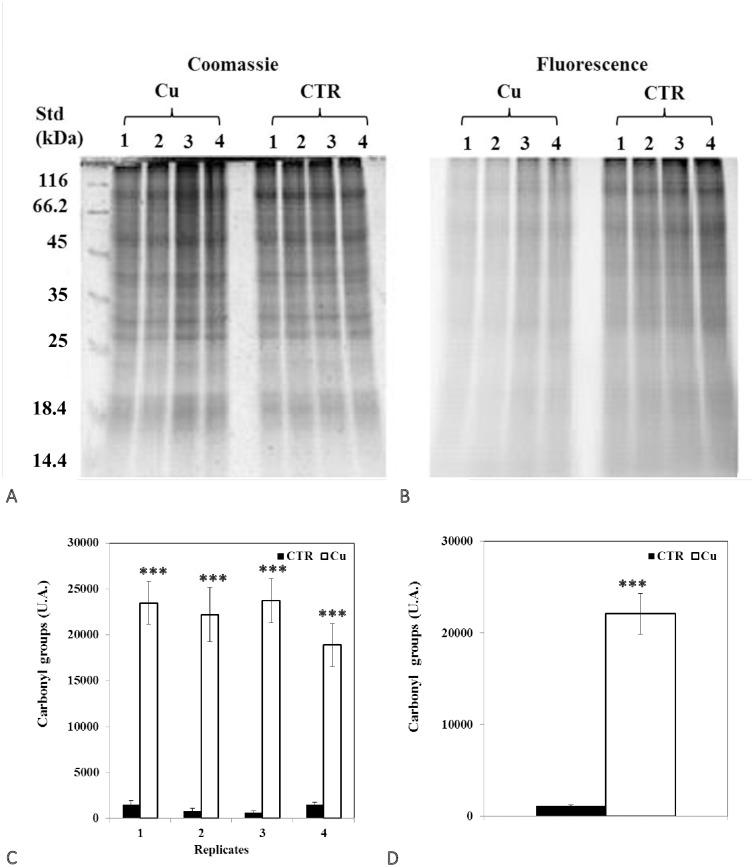
Representative images of 1DE gels of proteins (150 μg) in seedlings of bean seeds germinated for 9 days in (A) the presence of distilled water (CTR) or (B) 200 μM Cu. Gels were stained with Coomassie G-250 (scanned with GS-800 calibrated densitometer) and with FTSC labeling (scanned with Typhoon 9400 scanner). Total optical densities for each lane obtained from FTSC staining were normalized with those from Coomassie G-250 staining of the same gel. (C, D) Levels of proteins containing carbonyl groups. Values shown are means of (C) 4 biological replicates (±SD) numbered from 1 to 4, and means of (D) 4 technical replicates (±SD). Each measurement was performed in an extract obtained from several cotyledons. Analyses were performed using ANOVA, student’s T test; *p<0.05, ***p<0.001.

**Fig 8:**
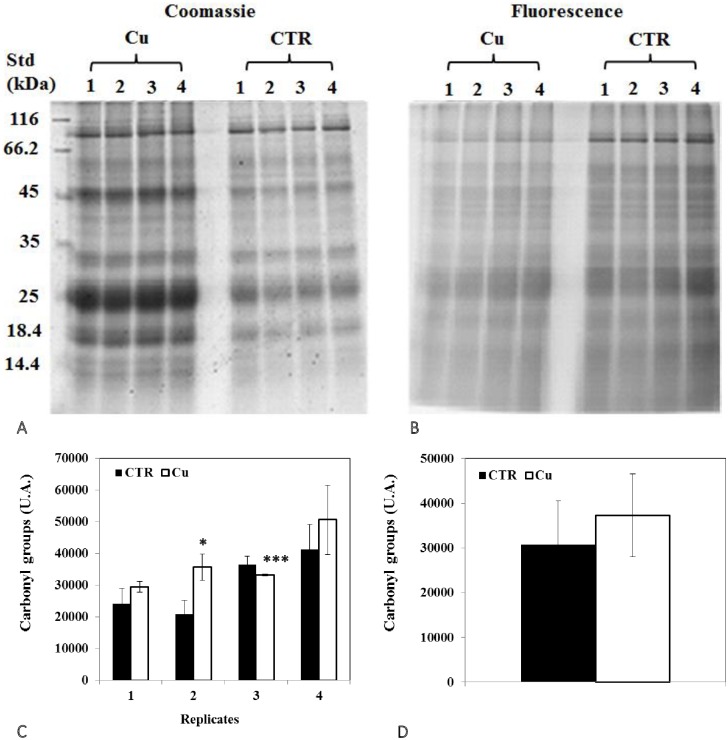
Representative images of 1DE gels of proteins (100 μg) in cotyledons of bean seeds germinated for 3 days in the presence of (A) distilled water (CTR) or (B) 200 μM Cu. Gels were stained with Coomassie G-250 (scanned with GS-800 calibrated densitometer) and with FTSC labeling (scanned with Typhoon 9400 scanner). Total optical densities for each lane obtained from FTSC staining were normalized with those from Coomassie G-250 staining of the same gel. (C, D) Levels of proteins containing carbonyl groups. Values shown are (C) means of 4 biological replicates (±SD) numbered from 1 to 4, and (D) means of 4 technical replicates (±SD). Each measurement was performed in an extract obtained from several cotyledons. Analyses were performed using ANOVA, student’s T test; ***p<0.001.

**Fig 9:**
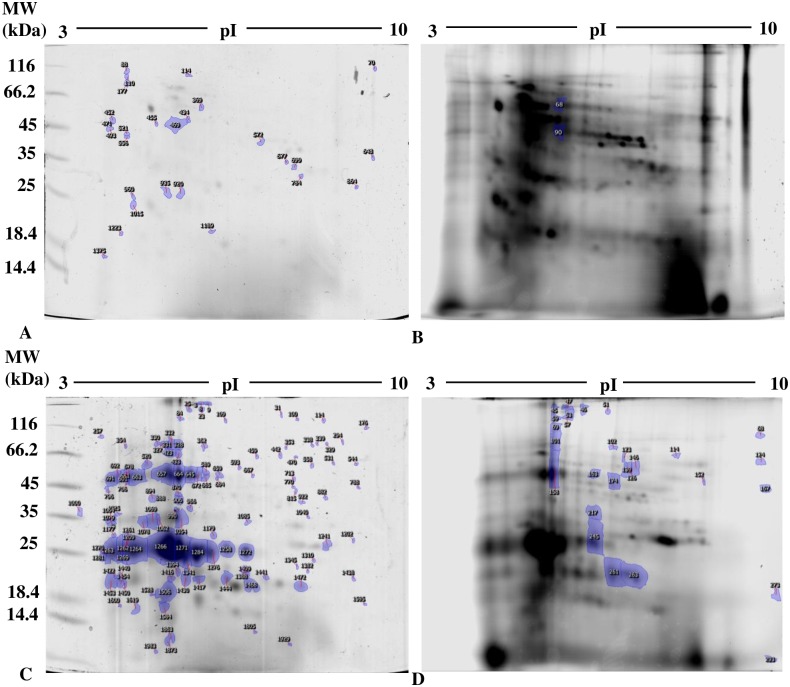
Profiles of the expression of proteins containing carbonyl groups in (A, B) seedlings and (C, D) cotyledons of bean seeds germinated for 9 days in the presence of distilled water (CTR) or 200 μM Cu. Proteins (1200 μg) were labeled with FTSC and separated by 2-D SDS-PAGE. Figures show spots of interest in representative gels from (A) colloidal Coomassie Brilliant G-250 staining (scanned with GS-800 calibrated densitometer) and (B) FTSC labeling (scanned with Typhoon 9400 scanner; 600 PMT). Numbers correspond to spots of p<0.05 and Fold induction >1.5 (spots identified).

Analysis of 1D SDS PAGE gels revealed a significant decrease in total protein thiols in seedlings (Fig 4), but an increase in cotyledons (Fig 5). In addition, representative 2D gel images of total proteins showed 1,174 and 599 spots, respectively, in seedlings and cotyledons (Fig 6; Table 5). Amongst these, 77 and 34, respectively, were significantly modified (±1.5-fold), compared to controls (p<0.05). It was noted in these preparations that the majority of proteins focused in the pI range 5–10 and in the M_r_ range 15–75 kDa. Comparison of spot patterns between Cu-treated and control samples revealed more increase than decrease of proteins, in the presence of Cu in both tissues, suggesting activation of biosynthesis upon heavy metal exposure. Fluorescence intensity measurements after IAF labeling showed 4 and 27 spots of interest, respectively, containing -SH groups (p< 0.05 and > = 1.5-fold). In cotyledons, all the proteins corresponding to 4 spots seemed to be increased in abundance whilst, in the seedlings, no significant variation was detected between replicates in the presence of Cu (13 increases *vs* 14 decreases, Fig 6). Figs 7 and 8 showed an increase in the total CO, respectively, in the seedlings and the cotyledons after Cu exposure. Carbonylation is known to be a general indicator of protein oxidation [6, 32]. These findings were corroborated by 2D gel analysis using FTSC-specific fluorescence. The representative 2D gels of CO groups of proteins showed 610 and 356 total protein spots, respectively, in cotyledons and seedlings. Among these, 234 and 159 corresponded with spots detected by fluorescence after FTSC labeling (Table 6). The interesting spots (significant at p<0.05 and > = 1.5 fold), 29 and 3 spots respectively, in cotyledons and seedlings (Fig 9) also showed more increase than decrease by Cu, suggesting enhanced protein biosynthesis under Cu-induced stress.

**Table 5:** Proteins containing thiol groups (IAF labeling) in the seedlings (3 days-old) (a) and the cotyledons (9 days-old) (b) of germinated bean seeds in the presence of H_2_O (CTR) or 200 μM Cu.

Number of spots	Coomassie	Fluorescence
Total	(a) 599	260
	(b) 1174	500
Significantly modified by Cu (p< = 0.05)	(a) 33	33
	(b) 92	7
Fold increase > = 1.5	(a) 33	27
	(b) 77	4
p< = 0.05, Fold increase > = 1.5	(a) 34	27
	(b) 77	4
Increased by Cu	(a) 20	13
	(b) 47	4
Decreased by Cu	(a) 14	14
	(b) 30	0

**Table 6:** Proteins containing carbonyl groups (FTSC labeling) in the seedlings (3 days-old) (a) and the cotyledons (9 days-old) (b) of germinated bean seeds in the presence of H_2_O (CTR) or 200 μM Cu.

Number of spots	Coomassie	Fluorescence
Total	**(a)** 356	159
	**(b)** 610	234
Significantly modified by Cu (p< = 0.05)	**(a)** 32	5
	**(b)** 130	31
Fold increase > = 1.5	**(a)** 27	3
	**(b)** 122	29
p< = 0.05, Fold increase > = 1.5	**(a)** 27	3
	**(b)** 122	29
Increased by Cu	**(a)** 19	1
	**(b)** 86	18
Decreased by Cu	**(a)** 8	2
	**(b)** 36	11

## Discussion

Because of their sessile character, plants confront various environmental stresses during their life cycles. Heavy metals, particularly Cu, represent a serious problem in agricultural production [48]. Research into germination and seedling growth (post-germination phase) are considered fundamental to evaluating the toxic effects of heavy metals on important agronomic plants such as beans [49]. It has been suggested that vulnerability of bean seeds towards copper stress can be partly explained by disruption of metabolic pathways affecting seedling growth [27, 28]. In the present work, a significant delay in seedling growth (Figs 1 and 2) was shown to be associated with metabolic disturbances possibly occurring in both seedlings and cotyledons. In fact, investigation of the changes in antioxidant metabolism and cellular redox status confirmed that Cu induced intrinsic production of ROS, notably H_2_O_2_ (Table 1). Many heavy-metal-stressed species have been reported to defend against ROS overproduction [50–52]. In the present work, the formation of H_2_O_2_ seems to be mediated by the redox-active Cu. Therefore, metal ions-catalyzed reactive oxygen radicals might be potent mediators of the cellular oxidative injury, which can damage proteins, nucleic acids, and lipids. Indeed, in addition to lipid peroxidation (see increased malondialdehyde levels in S1 Appendix), we aimed to investigate mainly changes affecting proteins. Cu exhibits an affinity for the sulfur in cysteine or methionine, and Cu^2+^ binds to oxygen or imidazole nitrogen groups of aspartic and glutamic acid, or histidine. In addition, Cu can displace other metals, such as zinc, from their cognate ligands in metalloproteins, which can result in inappropriate protein structures or inhibition of activity of many important cellular enzymes.

Additionally, generation of oxidative stress has been reported in germinating legume seeds after heavy metal exposure [53, 54]. Here, endogenous H_2_O_2_ accumulation, triggers stimulation of antioxidant enzymes SOD, CAT and peroxidases (APX, GPX and POX), thus allowing enhanced elimination of H_2_O_2_ in seedlings and cotyledon tissues after Cu exposure (Table 1; Fig 3). Enzymatic antioxidative response differs between seedlings and cotyledons, however, with respect to the order of activation of the antioxidative enzymes during germination (Figs 3 and 4). Changes in antioxidant concentrations and activities of ROS-processing enzymes have been associated with seed germination under heavy metals [55]. POXs are considered to be heavy-metal stress-related enzymes and are sometimes used as stress markers in metal poisoning scenarios [13]. Effects of heavy metals on antioxidant enzyme activities and their involvement in defense mechanisms against oxidative damage have been widely reported in the literature, but remain controversial and vary amongst plant species, different tissues and varying exposure regimes [3, 13, 53, 56]. Cu also inhibits some enzymes such as acid phosphatase (orthophosphoric-monoester phosphohydrolase, EC 3.1.3.2), G6PDH, isocitrate dehydrogenase, CAT, GPX and glutathione transferases. Antioxidant systems are likely to be involved in defense against heavy metal-imposed oxidative stress, but might also be direct biochemical targets for metallic ion-induced toxicity.

In addition to their enzymatic antioxidant capacity, plant tolerance to heavy metal-induced toxicity depends crucially on the availability of reduced cofactors, such as NAD(P)H [13, 14]. The key antioxidant and redox systems such as Trx, Grx and the Asc-GSH cycle depend heavily on NADPH rather than NADH for reducing equivalents. Hence, in response to oxidative stress, cells may need to shift from pathways producing NADH to others producing NADPH, such as the pentose phosphate pathway [15]. In this regard, increased activity of NAD(P)H-independent dehydrogenases, notably G6PDH, 6PGDH and MDH, in both seedlings and cotyledons (Table 1) after Cu treatment, most likely enable increased availability of NADPH to stressed cells [14, 54, 55].

In the current study, protein redox status and the major intracellular redox actors that control formation/reduction of intra- and/or inter-molecular disulfide bridges were also studied. Analysis of the components of different redox systems suggests that, in cotyledons, neither the Trx/NTR nor Grx/GR systems were involved in improving the protection of protein thiols to oxidation, possibly due to direct inhibition by Cu ions of the redox enzymes. Cu also seems to induce differential redox responses in cotyledons and seedlings. In fact, it seems that both Trx and Grx enzymes had not improved the redox status of thiols in cotyledons. However, in seedlings the levels of all components of the redox systems were elevated, thus suggesting a contribution of Trx/NTR/NADPH, despite the vulnerability of the coenzymes to enzymatic oxidation. A decreased level of reduced protein thiols was found coupled with increased carbonyl content in cotyledons (Table 2), indicating extensive protein oxidation [29]. But in seedlings, despite an increase in protein carbonyl content, enhanced protein thiol levels (Table 2) suggest that thiol status is protected via Trx and Grx activities (Table 3). Redox systems are thought to play fundamental roles in controlling plant redox and defense status when subjected to abiotic stress [18, 24, 25]. Indeed, Trx are involved in protection against oxidative damage by regeneration of Prxs and methionine sulphoxide reductases, allowing detoxification of various peroxides and protein repair [57]. Trx h can act as a hydrogen donor for the peroxiredoxin-1 Cys, which protects macromolecules in seedlings against oxidation during the early phases of imbibition [58]. In cotyledons, loss of Fd and FNR activities may generate intracellular oxidative stress whilst, in seedlings, the Fd/FNR system appears to be involved in modulation of redox status (Table 3). On the other hand, increased Prx activity in both seedlings and cotyledons after Cu exposure suggest an antioxidant role, most probably *via* POX activity using H_2_O_2_, peroxynitrite and hydroperoxides as substrates [59].

In response to Cu stress, high levels of oxidized coenzymes compared to reduced ones accumulated in seedling and cotyledon tissues (Table 4), despite increased NAD(P)H-independent dehydrogenase activities. This observation is most likely due to enhanced consumption of NADPH following the induction of NTR activity in cotyledons and both NTR and GR activity in seedlings. Another explanation could be stimulation of enzymes oxidizing reduced coenzymes. For example, enhanced NAD(P)H oxidase activity could result in elevation of oxidized forms of coenzymes at the expense of reduced ones, thus increasing NAD(P)^+^/NAD(P)H ratios [53].

Cu-induced biochemical disturbances in germinating bean seeds, including modulation of activities of antioxidant enzymes, could prevent oxidative damage. However, differential redox responses in cotyledon and seedling tissues suggest a major capacity of redox systems to prevent oxidation of protein thiols in seedlings in particular. Protein thiol status of seedlings was not affected by Cu with an apparent increase in the reduced SH pool (Tables 3 and 4). This could be explained by higher Trx and Grx activities on protein thiols, underlining these proteins’ fundamental rules in controlling seedling redox homeostasis (despite other antioxidative alterations also described here). Recycling of protein thiols also appears to occur mainly *via* redox systems (Trx/NTR), (Grx/GR), (Fd/FNR) and Prx, rather than *via* NADPH. These results are corroborated by the study of proteomic changes occurring to SH and CO groups of proteins in both cotyledon and seedling.

Simultaneous profiling of many proteins represents a novel way to compare dynamic responses towards heavy metals [60]. Proteomics is increasingly used to detect effects of environmental contaminants in ecotoxicology [61, 62]. 2DE analysis revealed significant changes in abundance of SH and CO groups of protein species in cotyledons and the seedlings of Cu-stressed bean seeds (Figs 6–9). Oxidative stress can trigger conformational changes making protein thiols more reactive towards cationic groups and modifying their susceptibility to oxidation, either by increasing their exposure to the matrix or by decreasing side-chain *pKa* values [63]. In addition, transitory oxidative stress may increase protection of thiols, e.g. by glutathionylation or formation of sulphenic acid [64]. Heavy metals disrupt the majority of cellular mechanisms as well as causing differential accumulation of proteins involved in the regulation of cellular redox status [65]. Decreased availability of the main proteins involved in important cellular processes has also been reported [66].

In the present study, we have profiled the role of a network of ROS-detoxifying enzymes in protecting bean seeds from Cu-induced stress. Whilst antioxidant protection mechanisms have an important role in Cu stress tolerance in both cotyledons and seedlings, we have discovered subtle differences in the two organs. Notably, we found a greater capacity for protein protection in seedlings compared to cotyledons.

One of the likely ways for ROS to interact with proteins is through thiol modification of cysteine residues, which can be oxidised to varying degrees triggering changes in protein conformation and activity [67]. Redox signalling in higher plants may include the activation of the mitogen activated protein kinase cascade, inhibition of phosphatases and activation of Ca^2+^ channels and Ca^2+^ -binding proteins [68], as well as effects of plant hormones on signalling networks [69]. Kranner and Seal proposed [70] a triphasic stress model of seed redox control under salt stress, whereby the ‘alarm’ phase involves stress perception and transduction through the ROS-RNS-hormone signalling network, post-translational modification of macromolecules and an altered transcriptome so that the protection and repair machinery become activated and upregulated, respectively, in response to the perception of a stress and / or the initial damage caused. Under continuing stress (time or severity), the ‘resistance’ phase is reached when sufficient gene products required for protection and repair are produced to maintain viability. The resistance phase includes inducible protection (e.g. upregulated antioxidants that protect macromolecules from further damage), repair mechanisms (e.g. synthesis of DNA repair enzymes), and the elimination of redundant cells that are damaged beyond repair.

On the other hand, our understanding of seed tolerance or resistance to heavy metal exposure is far from complete and future research should be directed to achieve a better understanding of the mechanisms by which they act or interact with biomolecules and metabolic pathways during early and post-germination phases. The present work suggests that differential responses within different organs of the same seed may be due to differential mechanisms that confer stress resistance. There might also be much interaction, with feedback loops between gene expression, transcription and translation as well as interconnections between the various biochemical pathways responsible for metal tolerance, such as those that define the redox hub comprising ROS, antioxidants and plant hormones. Once we have achieved a more complete understanding of the pathways that confer tolerance to salinity and drought, it may be possible to up- or down-regulate sets of genes until those required for salt tolerance, or more generally, stress tolerance, have been identified. Our approach has profiled profound biochemical changes associated with development of oxidative stress under environmental stress conditions. The data reported here provide novel insights that may lead to a broader understanding of molecular responses to Cu-induced stress in higher plants, and the resulting consequences for growth, development and enhanced agricultural productivity.

## Supporting information

S1 Appendix Levels of MDA in the seedlings (3 days-old) and the cotyledons (9 days-old) of germinated bean seeds in the presence of H_2_O (CTR) or 200 μM Cu. (DOCX)

## Abbreviations

6PGDH: 6-phosphogluconate dehydrogenase
AsA: ascorbate
Asc-GSH: ascorbate-glutathione cycle
APX: ascorbate peroxidase
CAT: catalase
CO: carbonyls
FTSC: fluorescein-5-thiosemicarbazide
FNR: ferredoxin-NADP oxido-reductase
G6PDH: glucose-6-phosphate dehydrogenase
GPX: glutathione peroxidase
GR: glutathione reductase
Grx: glutaredoxin
GSH: reduced glutathione
GSSG: oxidized glutathione
GST: glutathione transferase
IAF: 5’-iodoacetamide fluorescein
IEF: isoelectric focusing
MDH: malate dehydrogenase
MDHAR: monodehydroascorbate reductase
NTR: NADPH-dependent thioredoxin reductase
POX: peroxidase
ROS: reactive oxygen species
SDS-PAGE: sodium dodecyl sulphate-polyacrylamide gel electrophoresis
SH: thiols
SOD: superoxide dismutase
Trx: thioredoxin
