# Supplementary material for: Redox biology response in germinating Phaseolus vulgaris seeds exposed to copper: Evidence for differential redox buffering in seedlings and cotyledon
Source: PLoS One. 2017 Oct 5;12(10):e0184396. doi: 10.1371/journal.pone.0184396 (PMC5628808; doi:10.1371/journal.pone.0184396)
Supplement: S1 Appendix — (DOCX) [file pone.0184396.s001.docx]

**S Appendix.** Levels of MDA in the embryos (3 days-old) and the cotyledons (9 days-old) of germinated bean seeds in the presence of H_2_O (CTR) or 200 µM Cu. Values are means ± SE (n=4). Asterisks indicate significant differences compared with the respective control sample (*** p < 0.001). Procedure: samples homogenization in 20 mM Tris-HCl (pH 7.4, 1:5, w/v), then homogenates were centrifuged at 3000 ×g for 20 min and derivatized in the reaction mixture containing 10.3 mM 1-metyl-2-phenylindole (dissolved in acetonitrile/methanol, 3/1, v/v), HCl 32%, water and an equal volume of sample or 0-6 mM 1,1,3,3 tetramethoxypropane in 20 mM Tris-HCl (pH 7.4). After 40 min of incubation at 45 °C, samples were cooled on ice, centrifuged at 15,000 ×g for 10 min and the absorbance of the supernatant was recorded at 586 nm. Levels of MDA were calibrated against a malondialdehyde standard curve and expressed as nmol mg^-1^ protein.

**In embryo : CTR: 0.08±0.02 *versus* Cu: 0.20±0.05 *****

**In cotyledon : CTR: 0.11±0.08  *versus* Cu: 0.53±0.01 *****
